# Systemic inflammatory indices as a non-invasive grading modality for endometriosis: a comparative study versus exploratory laparoscopy

**DOI:** 10.61622/rbgo/2024rbgo84

**Published:** 2024-12-04

**Authors:** Ahmed Sabra Ibrahim Mohammed Sabra, Shreen Naguib Aboelezz Moselhy, Ahmed Kasem Mohamed Zain Eldin

**Affiliations:** 1 Benha University Faculty of Medicine Department of Obstetrics and Gynecology Benha Egypt Department of Obstetrics and Gynecology, Faculty of Medicine, Benha University, Benha, Egypt.

**Keywords:** Endometriosis, Neutrophils, CA125 antigen, Laparoscopy, Inflammation, Leukocyte count, Lymphocytes

## Abstract

**Objective:**

Included evaluation of the possibility of using the systemic inflammatory indices for preoperative screening for the presence and severity of endometriosis (EM) in comparison to the findings of the exploratory laparoscopy

**Methods:**

88 women with clinical manifestations suggestive of EM were evaluated clinically and by US and gave blood samples for estimation of serum cancer antigen-125 (CA125), platelet and total and differential leucocytic counts for calculation of inflammatory indices; the Systemic Immune-Inflammation index, the Systemic Inflammation Response Index (SIRI), the Neutrophil-Lymphocyte ratio (NLR), the Neutrophil-Monocyte ratio, the Neutrophil-Platelet ratio and the Platelet-Lymphocyte ratio. Then, patients were prepared to undergo laparoscopy for diagnosis and staging.

**Results:**

Laparoscopy detected EM lesions in 63 patients; 27 of stage I-II and 36 of stage III-IV. Positive laparoscopy showed significant relation with US grading, high serum CA125 levels, platelet and inflammatory cell counts and indices. Statistical analyses defined high SIRI and NLR as the significant predictors for positive laparoscopy and high serum CA125 and NLR as the most significant predictors for severe EM (stage III-IV) on laparoscopy

**Conclusion:**

The intimate relation between EM and inflammation was reflected systematically as high levels of blood cellular components, but indices related to neutrophil especially NLR and SIRI showed highly significant relation to the presence and severity of EM and might be used as routine, cheap and non-invasive screening test before exploratory laparoscopy to guide the decision-making.

## Introduction

Endometriosis (EMs) is one of the commonest chronic inflammatory disorder affecting women and is characterized by the presence and growth of endometrial-like glandular epithelial and stromal cells outside the uterus^(1)^ leading to multiple clinical symptoms affecting patients quality of life and fertility with high recurrence rate.^(2)^

Inflammatory cells are generic regulators of cancer with conflicting roles;^(3)^ macrophage and neutrophils are cancer promoting cells through the release of effectors that can promote tumor angiogenesis and proliferation, facilitate tissue invasion and metastatic dissemination,^(4)^ while innate immune cell types can produce tumor-killing responses.^(5)^ On reverse, tumors can induce inflammatory response through the release of chemotactic factors to recruit macrophages, damage-associated molecular patterns to activate granulocytes and neutrophils, and acidification of the tumor microenvironment to develop cancer-induced inflammatory response.^(6)^

The systemic immune inflammatory index (SII) and systemic inflammatory response index (SIRI) are two novel inflammatory biomarkers depending on the differential leucocytic count to use the counts of lymphocyte, neutrophil, monocyte and platelet counts for calculation of these indices.^(7)^

Both indices were documented as prognostic serum biomarker in many cancers and for the need and outcomes of adjuvant therapies for various cancers, where pre- and post-treatment SII and SIRI are associated with survival of patients with stage IV oropharyngeal cancers.^(8)^ High baseline SII and SIRI were associated with increased risk of recurrence of early-stage cervical cancer patients.^(9)^ Further, SII was identified as an independent predictor of abundance and maturity of tertiary lymphoid structure expression in non-small cell lung cancer.^(10)^

Considering the recent documentation for the predicative and prognostic ability of the systemic inflammatory indices in various cancers, the current study supposed possible applicability for these indices for prediction of EM disease severity as judged by exploratory laparoscopy.

The study aimed to evaluate the utility of the systemic inflammatory indices for preoperative prediction of EM disease severity in women assigned to exploratory laparoscopy for EM diagnosis and staging.

## Methods

All women attending the outpatient gynecology department with a clinical picture suggestive of having EM or had previously been diagnosed depending on radiologic workup were evaluated for the enrolment criteria.

Women with EM who were maintained on treatment or underwent operative interference during the last three months were not enrolled in the study. Also, patients who had autoimmune diseases, maintained on immunosuppressive therapy for any indication, cancer elsewhere in the body or receiving adjuvant therapies for cancer and patients receiving therapies for viral disorders especially COVID were excluded from the study. Moreover, patients refusing to undergo the exploratory laparoscopy were also omitted from the study.

Women with clinical manifestations suggestive of having EM or diagnosed clinically or by radiologic workup and were free of exclusion criteria were enrolled in the study.

All patients were subjected to the determination of their demographic data including age, body mass index (BMI), and evaluation for the presence of other diseases. Gynecological history taking for the symptoms suggestive of having EM especially pain as regards type, timing, and severity as judged by a visual analog scale (VAS) of 1-10 points with higher scores indicating more pain severity and the use of analgesia as regards its type. Menstrual and obstetric history data were also collected. General history taking with special regard to gastrointestinal and urinary manifestations suggestive of the possibility for intra-peritoneal spread of EM was obtained. Radiologic workups including abdomino-pelvic ultrasonography and MRI whenever indicated were undertaken.

The transvaginal sonographic examination was performed using a 7.5-MHz transvaginal probe for pelvic evaluation according to the 5-domain US-based approach as follows: routine assessment of the uterus and adnexa for the presence of adenomyosis or endometrioma (I), tenderness-guided assessment for possible peritoneal seedlings (II), assessment of the ovarian and uterine mobility to exclude or assure the presence of ovarian adhesions (IIIa) or obliterated Douglas pouch (IIIb) and the search for non-bowel (IV) or bowel (V) deep infiltrating EM.^(11)^

All patients received prophylactic broad-spectrum antibiotics with induction of general anesthesia. The patient was placed supine and a 1-1.5 cm just sub-umbilical incision was made along the skin crease, Verres needle was inserted to create pneumoperitoneum with a gradual elevation of abdominal pressure till 14 mmHg. Using the Storz endoscopic instruments (Karl Storz), a 10-mm trocar and telescope were inserted through the sub-umbilical incision and an accessory trocar was inserted lateral to the rectus sheath at about 4-5 cm above the anterior superior iliac spine. Then, the patient was positioned in the Trendelenburg position and exploratory laparoscopy was undertaken to visualize the ovaries, uterus, omentum, rectum and urinary bladder. Lesions were evaluated and staged and a biopsy was obtained whenever required to assure or grade the diagnosis. Lesions were classified according to the #Enzian classification as recently described by Keckstein et al.^(12)^

Blood samples were obtained under strict asepsis and were divided into two parts; one part was collected in EDTA-containing tubes and immediately sent to the hospital lab for determination of complete blood count including the differential leucocytic count. The other part was collected in plain tube, allowed to clot and centrifuged at 1500 rpm for 15 minutes and the resultant serum was frozen at −20^o^C till ELISA assayed for serum level of CA125 using Human CA125 ELISA Kit (Cat. No ab274402; Abcam Inc., San Francisco, USA).

Indices calculations

1. The Systemic Immune-Inflammation index (SII)
  SII was calculated using the equation proposed by Hu et al.^(13)^ as SII=PxN/L ; where P= platelet count, N= neutrophil count and L = lymphocyte count.
2. The Systemic Inflammation Response Index (SIRI)
  SIRI was calculated as SIRI=NxM/L ; where N= neutrophil count, M =monocyte count and L = lymphocyte count.^(14)^
3. The Neutrophil-Lymphocyte ratio (NLR)
  NLR was calculated as the resultant of dividing the neutrophil count by the lymphocyte count.^(15)^
4. The Neutrophil-Monocyte ratio (NMR)
  NMR resulted of dividing the total neutrophil count by the total monocyte count.^(16)^
5. The Neutrophil-Platelet ratio (NPR)
  NPR was calculated by dividing the neutrophil count by the platelet count.^(17)^
6. The Platelet-Lymphocyte ratio (PLR)
  PLR is the ratio between the absolute platelet and lymphocyte counts.^(18)^

Using IBM^®^ SPSS^®^ Statistics software (Version 22, 2015; Armonk, USA) the significance of differences between groups was assessed using the One-way ANOVA and Chi-square tests. Pearson's correlation analysis was applied to evaluate the relation between laparoscopic detection and severity staging of EM and US grades and inflammatory cell counts and inflammatory indices. The correlated variates were analyzed using the Regression analysis and the Receiver Operating Characteristic (ROC) Curve to define the predictors for the presence and severity of EM disease. The optimum cut off point for significance was *P*<0.05.

The study protocol was discussed with and approved by the departmental committee prior to start of case collection. Patients were fully informed by the items of the protocol before enrolment and patients accepted to participate in the study signed the informed consent. After complete case collection, the final approval was obtained by the Local Ethical Committee at 27.1.24. The study was registered at the clinicalTrial.gov ID: NCT06298617.

## Results

**Figure 1:**
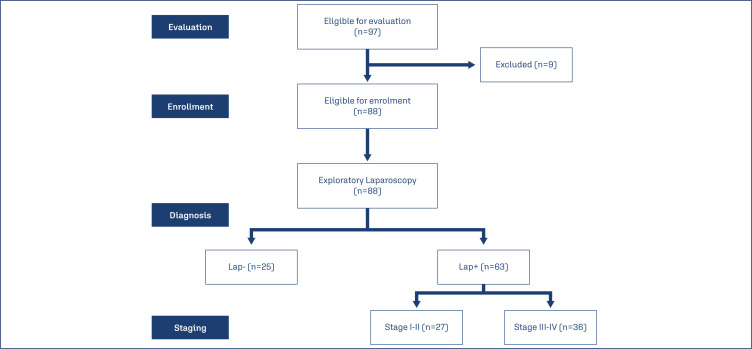
Patients’ selection process

During the study duration, 97 women were presented by clinical picture suggestive of having EM; 3 women had autoimmune diseases, 2 were maintained on medical therapy for EM, 2 patients underwent exploration for other causes and were unfit for abdominal insufflation, one patient had resection of endometrioma since one month and another refused to have the exploratory laparoscopy; these nine patients were excluded from the study. Eighty-eight patients were prepared for the exploratory laparoscopy that detected EM lesions of varied sites and severity in 63 patients (71.6%), while in the remaining 25 patients (28.4%) exploratory laparoscopy was considered negative and patients were categorized into Lap+ and Lap- groups. According to laparoscopic findings of patients of the Lap+ group, 12 patients (19%) were of Stage I, 15 patients (23.8%) had lesions of Stage II, 27 patients (42.9%) of Stage III and 9 patients (14.3%) had EM of stage V with a mean score of 2.5±1 (Figure 1). The enrolment data of patients of both groups as shown in table 1 showed in significant differences.

**Table 1:** Patients’ enrolment data

Variables		Lap- (n=25)	Lap+ (n=63)	p-value
Age (years)		31.4±5.1	33.4±4	0.055
Body mass index (kg/m^2^)		32.3±1.7	32±1.6	0.451
Family history of EM	Yes	3(12)	9(14.3)	0.778
	No	22(88)	54(85.7)	
Marital status	Single	11(44)	18(28.6)	0.286
	Married	13(52)	38(60.3)	
	Divorced	1(4)	7(11.1)	
Fertility of married or divorced women	Fertile	6(42.9)	16(35.6)	0.803
	1ry infertility	3(21.4)	13(28.8)	
	2ry infertility	5(35.7)	16(35.6)	
	Total	14(56)	45(71.4)	
Number of previous pregnancies among fertile and 2ry infertile women	One	6(54.5)	23(71.9)	0.178
	Two	3(27.3)	9(28.1)	
	Three	2(18.2)	0(0)	
	Total	11(78.6)	32(71.1)	
Number of living offspring	No	4(36.4)	7(21.9)	0.414
	One	4(36.4)	19(59.4)	
	Two	3(27.2)	6(18.7)	
	Total	11(78.6)	32(71.1)	

Pain is the main presenting symptom by all patients with insignificant differences between patients of both groups as regards the frequency of each type of pain. Some patients of the Lap+ group showed more than one type of pain with an incidence of 1.3 type of pain per patient and no patient of those who had negative laparoscopy complained of non-menstrual pelvic pain (NMPP). Scorings of dyspareunia were significantly (p=0.010) higher, while scorings for dysmenorrhea were insignificantly (p=0.075) higher among Lap+ than Lap- patients. Moreover, the mean value of the total pain score was significantly (p=0.0093) higher among patients of the Lap+ group. Patients’ distribution according to the type of the routinely used analgesia showed insignificant differences between patients of both groups. Fifty-three patients had manifestations other than pain with insignificantly higher frequency among Lap+ patients. Four Lap+ patients (6.3%) had dyschezia, two patients (3.2%) had dysuria, while 41 patients had pelvic and 6 patients had abdominal tenderness with no significant difference between both groups. US-imaging diagnosed 30 patients of Domain-I, 30 patients of Domain-II, and 15 patients of Domain-IIIa, while 13 patients of the Lap+ group were of Domain-IIIb, IV and V. Patients distribution among US domains was significantly higher among of Lap+ group. Also, scorings of US findings were significantly higher among patients of Lap+ than Lap- group (Table 2).

**Table 2:** Patients’ presenting data & US findings

			Lap- (n=25)	Lap+ (n=63)	p-value
Duration of symptoms (years)			3.2±1.7	3.8±1.8	0.132
Frequency of pain types	Dysmenorrhea		19(76)	52(82.5)	0.187
	Dyspareunia		6(24)	23(36.5)	
	NMPP		0(0)	9(14.3)	
	Type/patient		1	1.3	
Pain scores of each type of pain among the complaining patients	Dysmenorrhea		3.7±1.2	4.3±1.2	0.075
	Dyspareunia		3.5±1.1	5.04±1.3	0.010
	NMPP		0(0)	2.9±1.2	
Total pain score	Distribution	<5	19(76)	31(49.2)	0.065
		5-9	6(24)	30(47.6)	
		10	0	2(3.2)	
	Mean (±SD)		3.8±1.2	5.8±2.8	0.0093
Analgesia	Oral NSAID		18(72)	43(68.3)	0.102
	Injectable NSAID		9(36)	26(41.3)	
	Others		1(4)	9(14.3)	
	Type of analgesic/patient		1.12	1.24	
Other manifestations	Presence	No	22(35)	13(52)	0.139
		Yes	41(65)	12(48)	
	Type	Pelvic tenderness	31(49.2)	10(40)	0.528
		Abdominal tenderness	4(6.3)	2(8)	
		dyschezia	4(6.3)	0	
		Hematuria	2(3.2)	0	
US-evaluation		Domain I	16(64)	14(22.2)	0.0058
	Domain II		7(28)	23(36.5)	
	Domain III	IIIa	2(8)	13(20.6)	
		IIIb	0	3(4.8)	
	Domain IV		0	7(11.1)	
	Domain V		0	3(4.8)	
	Score		1.44±0.65	2.4±1.1	0.0001

NMPP - Non-menstrual pelvic pain; NSAID - Non-steroidal anti-inflammatory drugs

Estimated serum CA125 levels were significantly (p<0.001) higher in Lap+ patients who had positive laparoscopy than patients who had negative findings on exploratory laparoscopy. Despite the insignificantly lower hemoglobin concentration, the platelet and lymphocyte counts were significantly (p=0.008 & 0.0108, respectively) lower in patients of the Lap+ group than in patients of the Lap- group. The total leucocytic, neutrophil and monocyte counts were significantly higher in Lap+ patients than in Lap- patients, while basophil and eosinophil counts showed insignificant differences between both groups. The calculated NLR, NPR, SIRI and SII were significantly higher, while the calculated NMR and NPR were significantly lower in Lap+ than in Lap- patients (Table 3).

**Table 3:** Lap findings of the studied patients

		Lap- (n=25)	Lap+ (n=63)	p-value
Serum CA125 (IU/L)		26.64±18.1	100.3±72.1	<0.001
Hemoglobin concentration (g%)		11.82±0.6	11.5±0.9	0.112
Platelet count (10^3^/ml)		216.3±12.8	203±17.4	0.008
TLC (10^3^/ml)		6.89±0.73	7.7±1.28	0.0055
Differential leucocytes (cell/ml)	Neutrophil count	5020.6±633.7	5636.4±1312.4	0.0039
	Lymphocyte count	1262.8±155	1180±127.2	0.0108
	Monocyte count	319.2±70.6	390.8±96	0.0011
	Basophil count	169.3±44.8	186.9±37.3	0.077
	Eosinophil count	119.4±33.5	108±22.9	0.070
The inflammatory indices	NLR	4.01±0.55	5±1.24	0.0003
	NMR	19.5±4.9	16.84±7.29	0.097
	NPR	0.023±0.0033	0.029±0.0072	0.0004
	PLR	181.8±23.4	170.6±21.7	0.036
	SIRI	1087.4±315.5	1322.7±514.2	<0.001
	SII	866±120	1014.1±260.2	0.014

The detection of EM lesions during laparoscopy showed a significant relation with US grading, high serum CA125 levels, platelet count and inflammatory cell counts and indices. Moreover, all of these variates could predict or diagnose the positivity of the exploratory laparoscopy with significant AUC as evidenced by the ROC curve analysis. Univariate regression analysis defined high lymphocytic count and NLR ratio as highly significant (p<0.001) predictors for the possibility of finding endometriotic lesions on laparoscopy, while high monocytic count and serum CA125 as significant (p=0.009) predictor for positive laparoscopy for EM and high SIRI and high grade on US imaging are weakly predictors for positive laparoscopy for EM (P=0.016 & 0.021, respectively) as shown in table 4.

**Table 4:** Analyses for prediction of positive laparoscopy

Variates	Correlation analysis		ROC curve analysis				Univariate regression analysis	
	r	p-value	AUC	Std.	p-value	95% CI	β	p-value
US grading	0.402	<0.001	0.758	0.054	<0.001	0.653-0.864	0.179	0.021
CA125	0.477	<0.001	0.815	0.044	<0.001	0.729-0.901	0.213	0.009
Platelet	-0.350	0.001	0.294	0.054	0.003	0.187-0.400	-0.050	0.552
TLC	0.305	0.004	0.744	0.051	<0.001	0.645-0.844	0.054	0.741
Neutrophil	0.305	0.004	0.754	0.050	<0.001	0.656-0.852	0.052	0.747
Lymphocyte	-0.268	0.011	0.348	0.056	0.026	0.219-0.476	0.352	<0.001
Monocyte	0.342	0.001	0.740	0.055	<0.001	0.632-0.848	0.258	0.009
NLR	0.383	<0.001	0.864	0.039	<0.001	0.786-0.941	0.336	<0.001
NMR	-0.346	0.001	0.283	0.056	0.002	0.644-0.846	-0.037	0.735
NPR	0.341	0.001	0.745	0.051	<0.001	0.644-0.846	-0.023	0.870
PLR	-0.356	0.001	0.292	0.059	0.002	0.177-0.407	-0.220	0.082
SIRI	0.557	<0.001	0.862	0.039	<0.001	0.786-0.939	0.242	0.016
SII	0.334	0.001	0.753	0.050	<0.001	0.654-0.852	0.062	0.634

Multivariate Regression analysis for the significant variates on Univariate analysis defined high SIRI and NLR as the persistently significant predictors for positive laparoscopy while excluding the other variates (Table 5).

**Table 5:** Multivariate regression analysis

	Model 1		Model 2		Model 3		Model 4	
US grading	0.180	0.025	0.232	0.004	Excluded			
CA125	0.187	0.025	Excluded					
Lymphocyte	0.242	0.002	0.255	0.002	0.275	0.001	Excluded	
Monocyte	Excluded							
NLR	0.338	<0.001	0.354	<0.001	0.418	<0.001	0.350	<0.001
SIRI	0.412	<0.001	0.470	<0.001	0.470	<0.001	0.553	<0.001

The ROC curve analysis for the predictors of severe EM (stage III-IV) on laparoscopy defined high serum CA125 and NLR as the predictors of high significance (p=0.003 & 0.005, respectively), followed by high NPR (P=0.009), high SIRI and SII (p=0.014 & 0.019, respectively) and lastly high neutrophil count (p=0.037) as shown in table 6. Multivariate Regression analysis of these variates assured the predictability of high NLR for the presence of severe EM on laparoscopic evaluation (β=0.435, p<0.001) while excluding other variates.

**Table 6:** ROC analysis for prediction of EM severity according to laparoscopic grading

Variates	AUC	Std.	p-value	95% CI
CA125	0.765	0.073	0.003	0.622-0.908
TLC	0.571	0.083	0.435	0.409-0.733
Neutrophil	0.688	0.068	0.037	0.554-0.823
NLR	0.755	0.077	0.005	0.605-0.908
NPR	0.737	0.066	0.009	0.607-0.867
SIRI	0.723	0.096	0.014	0.535-0.912
SII	0.713	0.087	0.019	0.542-0.884

## Discussion

This study tried to evaluate the predictive ability of cellular blood components to identify patients who had EM out of those presenting by suggestive manifestations as a routine, cheap and non-invasive screening test before exploratory laparoscopy to help with decision-making. In line with this rationale, Kayacık Günday and Yılmazer^(19)^ found δ-neutrophil index, which is an inflammatory marker, and red cell distribution width, which is associated with inflammation, might be helpful to assure the clinically diagnosed EM to reduce the need for surgery.

The determined differential leucocytic counts and the calculated ratios showed significant differences between the Lap+ and Lap- patients. In hand with this result, multiple previous studies detected higher blood cellular counts and indices in blood samples of EM patients than counts detected in samples of controls^(20–26)^ and Jing et al.^(22)^ documented the greater sensitivity of the combination of NLR and CA125 to differentiate between endometriosis and benign ovarian tumors than CA125 alone. However, Moini et al.^(23)^ denied the ability of measurements of hematological parameters to diagnose EM.

In support of the obtained results concerning the ability of blood cellular indices to distinguish EM patients as documented by statistical analyses, Duan et al.^(27)^ documented that the reported positive correlation between NLR and PLR in blood samples of ovarian endometrioma patients confirms the role of inflammation in the pathogenesis of ovarian endometriosis and concluded that both ratios could be used as diagnostic biomarkers for EM. Furthermore, Tabatabaei et al.^(28)^ in a meta-analysis study found high NLR could identify EM patients from healthy controls and patients with other benign tumors and concluded that NLR might be applied as a possible predictor to help for diagnosis of EM

Moreover, statistical analyses of the evaluated blood markers and US findings for prediction of EM disease severity as judged and scored by laparoscopy defined high serum CA125 and NLR as the predictors of highest significance for differentiating patients who had severe EM (Stages III & IV) and regression analysis assured the high predictability of high NLR for that purpose. In line with these findings, Jing et al.^(22)^ reported a positive correlation between EM stage, oviduct adhesion, and diameter of ovarian ectopic cysts and both NLR and CA125.

Thereafter, Cho et al.^(29)^ found patients who had EM stage III/IV had significantly higher NLR than patients who had EM stage I/II, and those who had benign ovarian tumors documented that higher NLR than 2.5 might be an independent risk factor for severe EM disease. Also, Li et al.^(25)^ reported high AUC for PLR and SII for prediction of ovarian endometrioma staging and high SII has a predictive value for differentiation between endometrioma of stage III and IV, and that of stage I and II and found PLR, NLR, SII, SIRI and CA125 were significantly higher in patients with recurrence than those without recurrence during 2-years follow-up after surgery

In support of the role of inflammatory cells as a diagnostic modality for the presence and/or the severity of EM, Habata et al.^(30)^ using an animal model of EM found mesenchymal stem cells conditioned bone marrow allowed a twofold reduction of macrophages and neutrophils infiltrating the endometriotic lesions with a decrease of the proportion of M1/M2 macrophages, reduction of inflammation and suppressed expression levels of inflammatory markers with subsequent promoted tissue repair. Other studies used natural derivatives for the treatment of animals after induction of EM and found these materials reduced neutrophil infiltration, cytokines release through targeting the NOD-like receptor family pyrin domain containing 3 (NLRP3) inflammasome pathway,^(31)^ through reducing the number of macrophages and neutrophils with inhibition of the NF-κB signaling pathway^(32)^ or through the amelioration of fibrosis and adhesions via inhibition of neutrophils recruitment to endometriotic lesions and the production of monocyte chemoattractant protein-1 from neutrophils.^(33)^

Yang et al.^(34)^ suggested that inflammation through regulating the function of immune cells and promoting the activity of neutrophils, which are responsible for the secretion of proinflammatory cytokines, leads to increased levels of inflammatory and adhesion mediators that might mediate the adhesion, proliferation, differentiation, and invasion of ectopic endometriotic lesions. Also, Ajdary et al.^(35)^ conducted a therapeutic trial using dienogest; an oral progestin, and reported decreased levels of NLRP3, which is a cytosolic multi-protein complex responsible for the induction of inflammation with subsequent reduction of EM-induced pain.

Estimation of inflammatory mediators especially those secreted by neutrophils was a shortcoming of the study to evaluate the role of these mediators in EM severity.

Further studies are mandatory to establish the diagnostic role of NLR and SIRI for EM and to estimate the levels of inflammatory mediators in blood and peritoneal aspirate during laparoscopy or in tissue extract of the obtained specimens to evaluate the role of these mediators as diagnostic and prognostic biomarkers especially if the relation between their serum and tissue extract levels was established.

## Conclusion

The estimated neutrophils-related indices, especially NLR and SIRI showed highly significant relation to the presence and severity of EM and might be used as predictors to guide the decision-making.
